# Age, Weight, and *CYP2D6* Genotype Are Major Determinants of Primaquine Pharmacokinetics in African Children

**DOI:** 10.1128/AAC.02590-16

**Published:** 2017-04-24

**Authors:** Bronner P. Gonçalves, Helmi Pett, Alfred B. Tiono, Daryl Murry, Sodiomon B. Sirima, Mikko Niemi, Teun Bousema, Chris Drakeley, Rob ter Heine

**Affiliations:** a Department of Immunology and Infection, London School of Hygiene and Tropical Medicine, London, United Kingdom; b Department of Medical Microbiology, Radboud University Medical Center, Nijmegen, The Netherlands; c Department of Biomedical Sciences, Centre National de Recherche et de Formation sur le Paludisme, Ouagadougou, Burkina Faso; d Department of Pharmacy Practice, University of Nebraska Medical Center, Omaha, Nebraska, USA; e Department of Clinical Pharmacology, University of Helsinki and Helsinki University Hospital, Helsinki, Finland; f Department of Pharmacy, Radboud University Medical Center, Nijmegen, The Netherlands

**Keywords:** pharmacokinetics, primaquine, Plasmodium falciparum, CYP2D6

## Abstract

Low-dose primaquine is recommended to prevent Plasmodium falciparum malaria transmission in areas threatened by artemisinin resistance and areas aiming for malaria elimination. Community treatment campaigns with artemisinin-based combination therapy in combination with the gametocytocidal primaquine dose target all age groups, but no studies thus far have assessed the pharmacokinetics of this gametocytocidal drug in African children. We recruited 40 children participating in a primaquine efficacy trial in Burkina Faso to study primaquine pharmacokinetics. These children received artemether-lumefantrine and either a 0.25- or a 0.40-mg/kg primaquine dose. Seven blood samples were collected from each participant for primaquine and carboxy-primaquine plasma levels determinations: one sample was collected before primaquine administration and six after primaquine administration according to partially overlapping sampling schedules. Physiological population pharmacokinetic modeling was used to assess the impact of weight, age, and *CYP2D6* genotype on primaquine and carboxy-primaquine pharmacokinetics. Despite linear weight normalized dosing, the areas under the plasma concentration-time curves and the peak concentrations for both primaquine and carboxy-primaquine increased with age and body weight. Children who were CYP2D6 poor metabolizers had higher levels of the parent compound, indicating a lower primaquine CYP2D6-mediated metabolism. Our data indicate that primaquine and carboxy-primaquine pharmacokinetics are influenced by age, weight, and *CYP2D6* genotype and suggest that dosing strategies may have to be reconsidered to maximize the transmission-blocking properties of primaquine. (This study has been registered at ClinicalTrials.gov under registration no. NCT01935882.)

## INTRODUCTION

Since 2012, the World Health Organization (WHO) recommends the use of a single 0.25-mg/kg dose of primaquine (PQ) in combination with standard artemisinin-based combination therapy (ACT) for the treatment of Plasmodium falciparum malaria in elimination and resistance containment settings (1). The rationale for using PQ is to prevent transmission of malaria to mosquitoes since it is the only currently available antimalarial that accelerates the clearance of mature gametocytes post-ACT (2). Several recent trials assessed the efficacy of the WHO-recommended dose and concluded that it reduces gametocyte carriage compared to ACT alone and effectively prevents transmission in mosquito infection experiments (3–6). In addition to its use to prevent P. falciparum transmission as a single-dose treatment, PQ has been used for decades in multiple-dose regimens to clear Plasmodium vivax hypnozoites (7, 8).

The parent compound is not responsible for PQ effects on P. vivax hypnozoites (9) and P. falciparum gametocytes (10), and the drug-metabolizing cytochrome P450 2D6 (CYP2D6) enzyme has been implicated in the formation of unknown active metabolites that are responsible for the pharmacological effect of PQ (9, 11–13). In mice, knocking out the *CYP2D* locus can reduce the metabolism of PQ into its active metabolite against Plasmodium berghei (11) and increase the area under the plasma concentration-time curve for PQ (14). The gene coding for this enzyme (*CYP2D6*) is hypervariable in humans, and there is limited knowledge on the effects of the variation at this locus on the pharmacokinetics of PQ in humans (9). Early pharmacokinetic studies of PQ in adults (15–19) identified the main PQ metabolite, carboxy-PQ (C-PQ), which is slowly eliminated and is present at plasma concentrations up to 10 times higher than those of its parent compound (16). C-PQ is produced by monoamine oxidase (MAO)-A (12), an enzyme involved in drug metabolism in the liver (20), and indirect evidence (9, 21) suggests that it is not the active metabolite against malaria parasites or one of its precursors.

To date, there are only limited PQ pharmacokinetic data (16–18, 22). This is particularly evident for single low-dose PQ and for pharmacokinetic data in children: only one study, undertaken in Papua New Guinea and using single PQ doses of 0.5 or 1.0 mg/kg, recruited children (22). The difficulty to accurately dose children by extrapolating dosing schemes from adults (23–26) was previously illustrated for the antimalarials sulfadoxine-pyrimethamine and dihydroartemisinin-piperaquine (27). Since children are frequently infectious to mosquitoes (28) and comprise an important part of the human infectious reservoir for malaria (29), data on single low-dose PQ pharmacokinetics in children are highly needed for the planning of community treatment campaigns with PQ to reduce P. falciparum transmission. To identify factors that impact PQ pharmacokinetics in children, we performed a pharmacokinetic study of PQ in the largest pediatric population examined thus far.

## RESULTS

### Study population.

A total of 40 afebrile children aged 2 to 14 years who received a single low dose (0.25 or 0.40 mg/kg) of PQ on the final day of a six-dose artemether-lumefantrine (AL) regimen were included in this study. Of these 40, 37 had patent asexual-stage P. falciparum parasites at enrollment (median and interquartile range [IQR], 1,252 parasites/μl [578 to 2,503 parasites/μl]). The median (IQR) hemoglobin level at enrollment was 11.6 g/dl (10.8 to 12.5 g/dl) and similar in the two study arms. Table 1 summarizes the demographics and baseline laboratory results for these 40 children.

**TABLE 1:** Baseline characteristics

Parameter	Study arm (PQ dose)	
	0.25 mg/kg	0.40 mg/kg
No. of participants	20	20
Gender (% female)	55	60
Median (IQR)		
Age (yrs)	8 (6–10)	10 (6.5–12)
Body wt (kg)	19.8 (16.2–26.3)	24.9 (15.9–31.6)
Ht (cm)	118 (104–133.5)	129 (104–144.5)
Temp (°C)	36.6 (36.4–37.0)	36.7 (36.1–36.9)
No. of asexual parasites/μl	991 (635.5–2,066.5)	1,188 (284.5–3,340)
Hemoglobin (g/dl)^*a*^	11.4 (10.6–12.5)	11.9 (11.1–12.7)
Alanine transaminase (U/liter)	22.5 (17.5–34.5)	22 (17–29)
Aspartate transaminase (U/liter)	38 (32–46)	36 (27–41)
Total bilirubin (μmol/liter)	7.4 (5.4–9.9)	10.8 (8–13.8)
Creatinine (μmol/liter)	37.8 (35.5–40.8)	38.4 (32.7–45.2)

a Hemoglobin levels were measured by using Hemocue.

### *CYP2D6* genotyping.

*CYP2D6* genotyping was successful for 36/40 children. For 3/40 participants no samples were available for genotyping, and for one the genotyping was inconclusive. Allele frequencies are presented in Table S2 in the supplemental material. Of these 36 study subjects, 1/36 (2.8%), 10/36 (27.8%), 22/36 (61.1%), and 3/36 (8.3%) were classified as poor metabolizers (PM; activity score [AS] of 0), intermediate metabolizers (IM; AS of 0.5 or 1.0), extensive or normal metabolizers (EM; AS of 1.5 or 2.0), and ultrarapid metabolizers (UM; AS of 3.0), respectively (30, 31).

### Pharmacokinetics.

**FIG 1:**
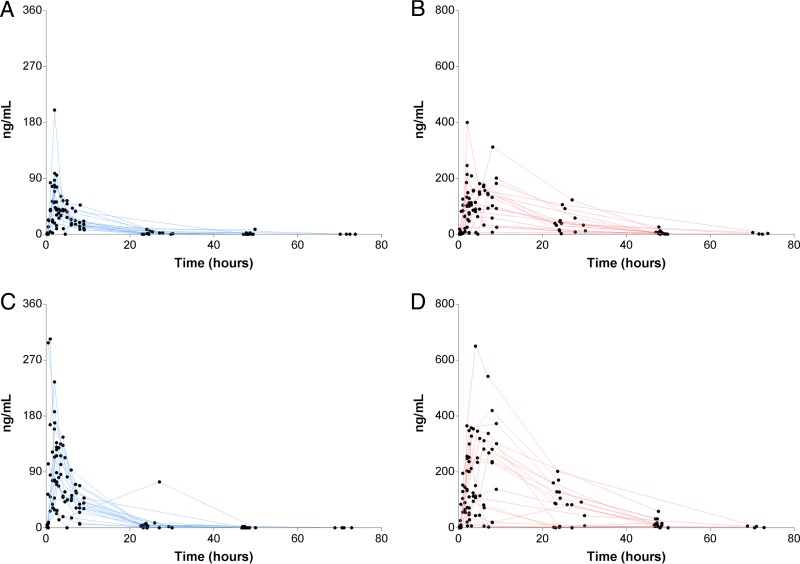
PQ and C-PQ plasma levels (*y* axes) after PQ administration (*x* axes). In panels A and C, PQ levels are presented for participants who received the 0.25- and 0.40-mg/kg PQ doses, respectively. In panels B and D, the C-PQ levels are presented for the 0.25- and 0.40-mg/kg PQ study arms, respectively. Assay results for all samples collected after PQ administration, including those with PQ or C-PQ levels below the limit of detection (i.e., with assigned level of 0 ng/ml), are presented.

A total of 274 plasma samples were collected. Two of the 40 children were excluded from the pharmacokinetic analysis because it was not possible to determine PQ and C-PQ levels in their samples due to inadequate sample volume. The raw pharmacokinetic data of PQ and C-PQ per dose group are depicted in Fig. 1. As observed, PQ was rapidly absorbed, and the plasma C-PQ concentrations were generally higher than the plasma PQ concentrations. Overall, the pharmacokinetics of the parent compound and of its main plasma metabolite presented substantial interindividual variation.

**FIG 2:**
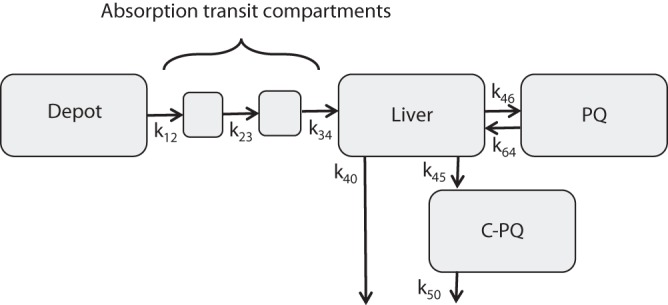
Schematic representation of the model. The mass transport of this model can be described with the following rate constants: *k*_12_ = 3/MAT, *k*_23_ = 3/MAT, *k*_34_ = 3/MAT, *k*_40_ = CL_H,CYP2D6_/*V*_L_, *k*_45_ = CL_H,MAO_/*V*_L_, *k*_50_ = CL_CPQ_/*V*_CPQ_, *k*_46_ = [*Q*_H_ (1 − *E_H_*)]*/V*_L_, and *k*_64_ = *Q*_H_/*V*_PQ_.

**FIG 3:**
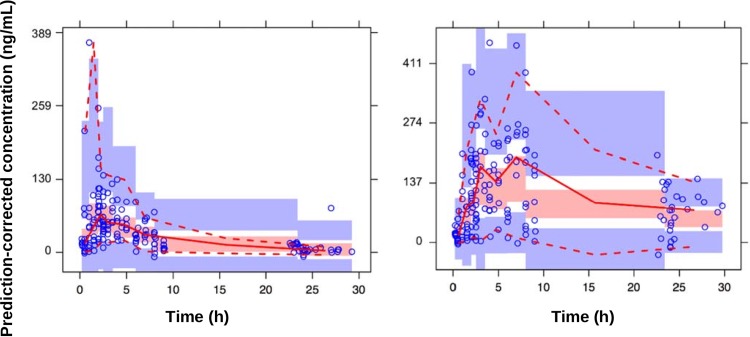
Prediction-corrected visual predictive check of observed data. The left and right panels depict the prediction-corrected visual predictive checks for PQ and C-PQ, respectively, based on 1,000 simulations. Prediction-corrected simulated (shaded areas) and observed (circles and lines) PQ and C-PQ concentrations are presented over time (h; *y* axes). The thick red line connects the observed median values per bin. The dotted red lines connect the 5th and 95th percentiles of the observations. The light blue areas are the 95% confidence interval of the 5th and 95th percentiles, and the light red area indicates the confidence interval of the median.

A physiological pharmacokinetic model was developed to estimate pharmacokinetic parameters for both PQ and C-PQ (Fig. 2). First-order kinetics with two absorption transit compartments and one compartment disposition for PQ and C-PQ fit the observed plasma levels well. PQ was rapidly absorbed with a mean absorption time of 0.706 h (relative standard error [coefficient of variation], 12%). Although allometric clearance appeared to explain most weight-related variability in pharmacokinetics, overprediction of plasma PQ and C-PQ concentrations was observed in the youngest children of our study population. This resulted in higher than expected estimates for apparent volume of distribution and apparent clearance in these children. This phenomenon may be explained by a reduced relative bioavailability at younger age. Therefore, maturation of relative bioavailability (*F*) with age (i.e., the increase in bioavailability with age) was described by an *E*_max_ model according to the following formula: *F* = age/(age + *F*_50_), where *F*_50_ is the age in years at which the relative bioavailability is 50% that of the mature value. *F*_50_ was estimated to be 4.27 years (relative standard error, 44%), explained all observed interindividual variability in relative bioavailability, significantly (*P* < 0.001) improved the model fit, and was therefore retained in the model. In addition to apparent age-dependent bioavailability, the inclusion of CYP2D6-mediated clearance of PQ, assumed to be linearly related to the CYP2D6 activity score, significantly improved model fit (*P* < 0.001) and was also retained in the final model. Diagnostic prediction-corrected visual checks of the model are shown in Fig. 3 and additional goodness-of-fit assessments are presented in Fig. S1 and S2 in the supplemental material. Estimated model parameters and their variability are presented in Table 2.

**TABLE 2:** Pharmacokinetic model parameters^*a*^

Parameter	Estimate	Relative SE of estimate (% CV)	Shrinkage (%)
Mean absorption time (h)	0.706 h	12	
PQ vol of distribution (*V*_PQ_) (70 kg), liters	127	20	
*V*_PQ_ interindividual variability (%)	82.9	38	12.2
C-PQ vol of distribution (*V*_CPQ_) (70 kg), liters	21.7	39	
CL_int,MAO_ (70 kg), liters/h	7.35	41	
CL_int,MAO_ interindividual variability (%)	65.3	33	13.1
CL_int,CYP2D6,pop_ (70 kg), liters/h	6.70	69	
CL_CPQ_ (70 kg), liters/h	1.50	35	
*F*_50_ (yrs)	4.27	44	
Residual error: PQ			
Proportional (%)	32.7	42	19.5
Additive (ng/ml)	2*		
Residual error: CPQ			
Proportional (%)	45.2	20	21.6
Additive (ng/ml)	2*		

a All flow and volume parameters were allometrically scaled to a body weight of 70 kg with an allometric exponent of 0.75 for flow parameters and an exponent of 1 for volume parameters. CV, coefficient of variation. *, values fixed during modeling.

The model-derived median (range) area under the concentration-time curve (AUC), the maximum concentration (*C*_max_), and the time of *C*_max_ (*T*_max_) for PQ were 600.26 h·ng/ml (259.87 to 3,315.40 h·ng/ml), 68.42 ng/ml (20.21 to 391.16 ng/ml), and 1.59 h (0.96 to 2.12 h), respectively, and are in agreement with previous studies (18); for C-PQ these values were 3,468.35 (962.05 to 10,506) h·ng/ml, 147.23 (25.61 to 403.95) ng/ml and 6.80 (2.73 to 16.03) h, respectively. The 0.40-mg/kg PQ dose was associated with a higher AUC and *C*_max_ compared to the 0.25-mg/kg dose (Table 3), although there was substantial variation within each study arm: *C*_max_ estimates included values that were 6 to 10 times higher than the lowest model-derived *C*_max_ in each study arm; a similar pattern was observed for the AUC.

**TABLE 3:** Model-derived pharmacokinetic parameters for PQ and C-PQ in African children receiving a single PQ dose of 0.25 (*n* = 18) or 0.40 (*n* = 20) mg/kg

Parameter	Median (range)			
	0.25 mg/kg (PQ dose)		0.40 mg/kg (PQ dose)	
	PQ	C-PQ	PQ	C-PQ
*T*_max_ (h)	1.6 (1.2–1.9)	7.1 (4.3–10.1)	1.6 (1.0–2.1)	6.6 (2.7–16.0)
*C*_max_ (ng/ml)	50.2 (20.2–138.7)	108.3 (25.6–240.2)	88.9 (24.4–391.2)	196.8 (29.3–404.0)
AUC_0–∞_ (h·ng/ml)	450.4 (259.9–875.9)	2,912.3 (962.1–5,076.3)	730.7 (334.2–3,315.4)	5,091.7 (1,075.0–10,506)

### Host characteristics influencing PQ pharmacokinetics.

**FIG 4:**
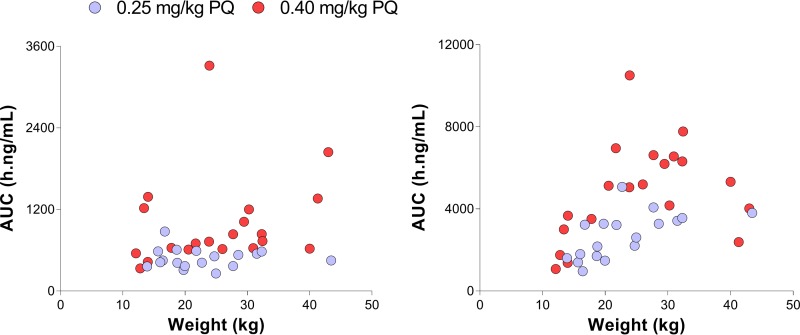
Area under the plasma concentration-time curves for PQ (left panel) and C-PQ (right panel) by weight (*x* axes).

PQ doses are linearly scaled with body weight. To assess whether this approach is appropriate, we analyzed plasma PQ and C-PQ concentrations by weight. In Fig. 4, the distribution of model-derived AUC values by weight and PQ dose is presented. For each dose, despite linear dose extrapolation based on weight, AUC estimates of PQ and C-PQ were positively correlated with body weight. A similar pattern was observed when analyzing the relationship between AUC values and age (see Fig. S3 in the supplemental material).

**FIG 5:**
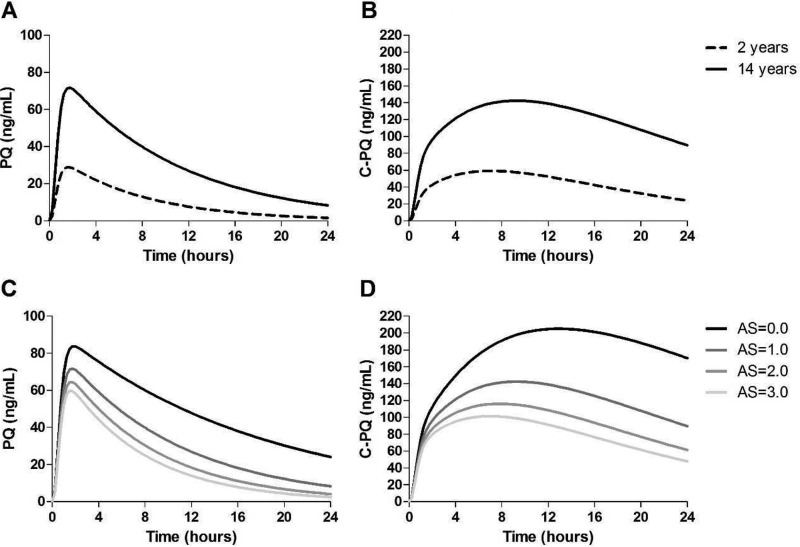
Effect of age and CYP2D6 activity score (AS) on PQ and C-PQ plasma concentrations over time after a 0.25-mg/kg single dose of PQ. Panels A and B, respectively, show the effects of age on PQ and C-PQ concentrations over time in two hypothetical children of different ages (2-year-old, 12-kg body weight; 14-year-old, 40-kg body weight), with a CYP2D6 AS = 1.0. Panels C and D, respectively, show the effects of genetically determined CYP2D6 AS in four hypothetical 14-year-old children on the PQ and C-PQ concentration over time. The values used to construct curves are model derived.

CYP2D6 AS was an important determinant of PQ and C-PQ concentrations. In Fig. 5, typical plasma PQ and C-PQ concentration-time curves for children aged 2 (body weight, 12 kg) and 14 (body weight, 40 kg) years old and with different CYP2D6 metabolizer status are presented. Toddlers who are IM have particularly low plasma levels of PQ compared to schoolchildren with PM status.

## DISCUSSION

The use of PQ to prevent P. falciparum transmission may support malaria elimination activities and efforts to contain artemisinin resistance. Understanding PQ pharmacokinetics is important to optimize a dosing regimen aimed at clearing gametocytes and reducing infectivity to mosquitoes. We performed the first PQ pharmacokinetic study in African children and observed that age, body weight, and *CYP2D6* genotype influenced PQ and C-PQ plasma levels: younger children and children with lower body weight have lower levels of PQ and C-PQ, whereas poor CYP2D6 metabolizers have higher levels of PQ. These findings indicate that linear weight-based dosing may be suboptimal to achieve efficacious PQ concentrations.

Since the 1980s several PQ pharmacokinetic studies have been performed; however, only one of these studies enrolled children (22). This is the first PQ pharmacokinetic study recruiting African children, a group that may represent 28.4 to 51.8% of all individuals capable of infecting mosquitoes in some endemic areas (28) and thus are an important target population for PQ treatment. Our analysis generated several insights into PQ pharmacokinetics in this age group. First, both age and weight explain variability in PQ pharmacokinetics in children and show a nonlinear relationship with PQ exposure. This is illustrated by the clear relationship between age and PQ exposure despite linear dose normalization based on weight. Therefore, linear dosing based on total body weight is inappropriate to obtain similar drug exposure in children as in adults, confirming earlier findings that (nonlinear) allometric scaling of pharmacokinetics of PQ accounted for the observed variability in Papua New Guinean children (22). However, this study did not report an age effect on bioavailability as observed by us. This might be explained by the fact that in our study PQ was administered using 1 mg/ml solutions, which allows for more accurate dosing than tablet-based regimens, and that our study population covered a wider age range (2 to 14 years) compared to the previous study in Papua New Guinea (6 to 10 years). The physiological cause of the observed maturation of bioavailability remains unclear. This phenomenon is not uncommon in pediatric pharmacokinetics: for example, the bioavailability of the liquid formulation of the antiretroviral drug efavirenz also matured with age in young children (32). Potential causes could be age-related changes in gastrointestinal motility, pH, or prehepatic expression of metabolic enzymes or transporters, possibly resulting in increased absorptive capacity with age (33, 34). These findings should be confirmed prospectively with additional PQ pharmacokinetic and pharmacodynamic studies in pediatric, as well as adult populations, to assess the effect of weight and age on PQ-related pharmacokinetics, as well as gametocyte clearance, transmission reduction, and toxicity.

Another important finding of our study is that *CYP2D6* genotype influences PQ pharmacokinetics (14). Initial PQ concentrations are affected by the *CYP2D6* genotype since CYP2D6-mediated metabolism occurs both systemically and presystemically. The influence of CYP2D6 metabolizer status on PQ efficacy was previously shown by an increased P. vivax relapse rate in individuals with *CYP2D6* PM and IM genotypes (9). Individuals with higher relapse rates, presumably because of an inability to clear P. vivax hypnozoites, had a significantly higher PQ AUC than extensive metabolizers but no differences in C-PQ pharmacokinetics parameters, which suggests that CYP2D6 activity is a rate-limiting step in the formation of active metabolite(s) against P. vivax hypnozoites (9, 11). It has been hypothesized that unknown metabolites formed through CYP2D6 are also responsible for the effect of PQ on P. falciparum transmission; however, direct evidence for this is lacking, and the available field studies with comprehensive CYP2D6 data are too small to assess the effect of CYP2D6 metabolizer status on gametocytemia and transmission potential after single low-dose PQ. CYP2D6-related differences in PQ transmission blocking efficacy would be particularly relevant in malaria elimination settings, where mass drug administration are used to accelerate transmission interruption: CYP2D6 poor metabolizers might remain infectious for longer periods of time after PQ administration compared to individuals with other *CYP2D6* genotypes and could represent a source of residual malaria transmission in these areas, depending on the frequency of alleles linked to this phenotype in the population.

Since PQ metabolism is considered essential to its effect on P. falciparum transmission (10), one may argue that exposure to the parent drug (PQ) is not important. However, since the formation of active metabolites depends on the presence of the parent drug, one should aim for an adequate initial exposure of PQ. Our findings suggest that age, body weight, and *CYP2D6* genotype can all influence PQ levels and consequently may determine the levels of active metabolites generated. These observations and the fact that currently available PQ tablet sizes are not optimal for pediatric dosing, posing further challenges in achieving the target PQ dose in children, indicate that PQ dosing strategies may have to be reconsidered. The therapeutic range over which PQ prevents malaria transmission is currently not well established but may include doses lower than the WHO-recommended PQ dose of 0.25 mg/kg (5). PQ efficacy and added value over ACT alone may also depend on the ACT used (4, 5, 35). The gametocytocidal and transmission blocking effects of AL are superior to that of dihydroartemisinin-piperaquine, but there are concerns for drug-drug interactions between AL and PQ. Indeed, although artemether does not influence PQ metabolism (36), lumefantrine inhibits CYP2D6 *in vitro* (37, 38). In our study, although participants were treated with lumefantrine, we found that the CYP2D6 activity score explained the variability in PQ pharmacokinetics, indicating that if CYP2D6 was indeed inhibited by lumefantrine, this inhibition was incomplete at the time of PQ administration. Since AL alone is by far the most widely used ACT, our CYP2D6-related findings are of immediate relevance for ACT-PQ policies, and our findings of age-dependent exposure to PQ and C-PQ are likely to be independent of the type of ACT-PQ combination.

PQ is considered a valuable tool to support malaria elimination efforts. Whether it is deployed during mass drug administration campaigns, when all individuals in a community receive treatment, or during treatment of symptomatic falciparum malaria episodes, a substantial proportion of PQ doses are likely to be given to young children. Age-related changes in PQ pharmacokinetics, such as the maturation of bioavailability or a nonlinear change in clearance with weight, indicate that dose extrapolation from adult regimens based solely and linearly on weight may not be an optimal approach. A limitation of our study is that the studied population was relatively small and did not include subjects outside the 2- to 14-year range. Extrapolation of our findings outside this range should, therefore, be performed with caution. Future studies relating plasma concentrations of PQ and its (active) metabolites are needed to quantify the implications of our findings for the ability to prevent P. falciparum transmission by PQ treatment and to inform better dosing strategies.

## MATERIALS AND METHODS

### Study site, approvals, and patients.

A randomized placebo-controlled trial to assess the effect of low-dose PQ on malaria transmission was undertaken in Balonghin, a village with endemic malaria transmission in Burkina Faso. Study procedures and results were described in detail elsewhere (4). Briefly, parasitemic children aged between 2 and 15 years without malaria symptoms (no measured fever, reported fever, or anemia) and with normal glucose-6-phosphate dehydrogenase enzyme activity were recruited and treated with AL alone, with AL and a 0.25-mg/kg PQ dose, or with AL and a 0.40-mg/kg PQ dose. PQ dosing was achieved by crushing a 15-mg PQ tablet and preparing 1-mg/ml solution by dissolving the crushed tablet in 15 ml of water. This allowed precise dosing: the mean difference between the actual dose given and the assigned dose was −0.004 mg (95% confidence interval −0.006 to −0.001 mg) of PQ/kg of body weight for participants included in this pharmacokinetic study. AL was given twice daily over 3 days, and PQ or placebo was administered with the fifth AL dose. A subset of study subjects not included in mosquito membrane feeding experiments was invited to participate in the pharmacokinetic study. To minimize the number of blood samples taken per participant while maximizing the number of time points with information on PQ and C-PQ levels, partially overlapping sampling schedules were designed, and sampling times were sequentially allocated to participants. The exact time when each blood sample was collected was recorded and used in pharmacokinetic analyses. A total of seven 1.5- to 2-ml venous blood samples were collected for each study subject: one sample before PQ or placebo administration, four in the first 12 h following this dose, and two between 24 and 72 h. All samples were centrifuged within 2 h of collection, and plasma was subsequently stored at −80°C. Forty participants, 20 from each PQ study arm, had PQ and C-PQ plasma levels quantified and were included in this analysis.

The study was registered at ClinicalTrials.gov (reference number NCT01935882). Written informed consent was obtained for participation in the pharmacokinetics sampling. The clinical trial received ethics approval from the London School of Hygiene and Tropical Medicine ethics committee (reference 6274) and the Comité d'Ethique pour la Recherche en Santé (Ministère de la Santé du Burkina Faso; reference 2012-10-78).

### Quantification of PQ and C-PQ plasma levels.

PQ and C-PQ levels were determined by liquid chromatography-mass spectrometry (LC-MS) as previously described (39). The system consisted of a Shimadzu LCMS-2010A mass spectrometer operated using electrospray ionization (ESI) in positive ion detection mode. Data were collected in the selected ion monitoring mode at 325.35 *m/z* for quinine (internal standard; retention time, 3.7 min), 260.30 *m/z* for PQ (retention time, 5 min), and 275.25 *m/z* for C-PQ (retention time, 8 min). The analytical column was a Phenomenex Synergi Polar RP (150 by 2 mm, 4 μm), preceded by a Polar RP security guard column (2 by 4 mm; Phenomenex, Torrance, CA). The standard curve ranged from 4 to 1,000 ng/ml, with a lower limit of quantitation of 4 ng/ml and a lower level of detection of 1 ng/ml. All control values were within 15% of their nominal value.

### *CYP2D6* genotyping.

EDTA-anticoagulated venous blood and/or saliva samples collected with an Oragene kit (OG-500 or OG-575) were used as sources of human genomic DNA for *CYP2D6* genotyping. DNA was extracted using a MagNAPure LC automated extractor and extraction kits for large volume samples according to the manufacturer's instructions. DNA concentration was measured fluorometrically using a Qubit fluorometer and the accompanying high-sensitivity kit. Samples were diluted according to the manufacturer's instructions for assays determining copy number variation (CNV) and for preamplification, as well as sequence variant determination with QuantStudio 12K Flex OpenArrays with TaqMan assays. OpenArray analysis was repeated for five samples without preamplification due to an undetermined genotype. In total, two CNV assays (hs00010001_cn targeting exon 9 and hs04083572_cn targeting intron 2 of *CYP2D6*) and 19 sequence variants in *CYP2D6* were analyzed (see Table S1 in the supplemental material for assay details) and genotype was determined according to the cytochrome P450 allele nomenclature website (40). Inferred phenotype was determined using the activity score (AS) (41).

### Pharmacokinetic modeling.

A physiological population pharmacokinetic model was developed to allow better extrapolation and enable identification of pharmacokinetic parameters that cannot be identified in classical empirical models (42). Pharmacokinetic analysis was performed by means of nonlinear mixed effects modeling with the software NONMEM v7.3.0 and using Piraña as an interface for NONMEM, R-statistics, and Perl Speaks Nonmem v4.6.0 (43). The covariance step in NONMEM was used to calculate parameter precision. To account *a priori* for changes in pharmacokinetics related to growth, liver volume (*V*_L_) was calculated from total body weight and height (44). All other volumes and flow parameters were allometrically scaled to a total body weight of 70 kg, as previously proposed. The exponents of the allometric models were fixed at 0.75 and 1 for flow and volume parameters, respectively (45). Due to the high colinearity of age and weight, this enabled us to separately assess the impact of age and weight on PQ pharmacokinetics. The PQ dose and measured plasma concentrations of PQ and C-PQ were converted to their molar equivalents for this analysis. Parameter shrinkage, with a shrinkage of >25% indicating uninformative data to estimate the parameter (46), was derived from the NONMEM results file.

As PQ is thought to be mainly metabolized by MAO and cytochrome P450s in the liver (12), a well-stirred liver model, a well-established model to describe hepatic metabolism of drugs, was implemented to describe the physiologically plausible relationship between first-pass and central metabolism (47, 48). Apparent intrinsic hepatic clearances for MAO- and CYP2D6-mediated metabolism (CL_int,MAO_ and CL_int,CYP2D6_, respectively) were estimated. C-PQ was assumed to originate from the MAO-mediated metabolism of PQ. The individual CYP2D6 intrinsic clearance (CL_int,CYP2D6,i_)was calculated from the population intrinsic clearance (CL_int,CYP2D6,pop_) and CYP2D6 AS according to the following formula: CL_int,CYP2D6,i_ = AS × CL_int,CYP2D6,pop_. We assumed a liver plasma flow (*Q*_H_) of 49.5 liters/h, derived from an adult total blood flow of 90 liters/h and a plasma fraction of 55% in whole blood (hematocrit level, 45%). The hepatic extraction (*E*_H_) was defined as follows: *E*_H_ = CL_int_/(*Q*_HP_ + CL_Int_), and the apparent MAO- and CYP2D6-mediated hepatic clearances (CL_H,MAO_ and CL_H,CYP2D6_) were calculated using the following formula: CL_H_ = *E*_H_ × *Q*_HP_.

The gradual onset of oral drug absorption was described with a chain of transition compartments, as described earlier (49). In short, the mean absorption time (MAT) was estimated and the rate constant (*k*_tr_) for these transition compartments was calculated using the equation *k*_tr_ = (*n* + 1)/MAT, where *n* equals the number of transition compartments. The interindividual variability was modeled by means of an exponential variance model. Throughout model building, basic goodness-of-fit plots and prediction-corrected visual predictive checks (50) were explored. Concentrations that were below the limit of quantification (LOQ) were retained in the analysis employing the M6 method, as proposed by Beal (51), so the first concentrations below the limit of quantification were fixed to 1/2 LOQ, and a fixed residual additive error of 1/2 LOQ was introduced in the model. Individuals with missing or inconclusive *CYP2D6* genotype data (*n* = 4) were retained in the model by imputing the individual activity scores using mixture modeling, based on the frequencies observed in the study population, as proposed earlier (52). The Bayesian imputed activity score was estimated to be 1.5 for all four children. Since fixing the individual activity scores manually resulted in better model stability and no significant change of model goodness-of-fit, these missing activity scores were manually set to 1.5 in the final model. The final model was used to obtain the empirical Bayes estimates for the AUC to infinity, the *C*_max_, and the *T*_max_ for both PQ and C-PQ.

## Supplementary Material

Supplemental material
