# Supplementary material for: Age, Weight, and CYP2D6 Genotype Are Major Determinants of Primaquine Pharmacokinetics in African Children
Source: Antimicrob Agents Chemother. 2017 Apr 24;61(5):e02590-16. doi: 10.1128/AAC.02590-16 (PMC5404566; doi:10.1128/AAC.02590-16)
Supplement: Supplemental material [file supp_61_5_e02590-16__index.html]

Age, Weight, and CYP2D6 Genotype Are Major Determinants of Primaquine Pharmacokinetics in African Children — Supplemental material 

# Age, Weight, and *CYP2D6* Genotype Are Major Determinants of Primaquine Pharmacokinetics in African Children

## Supplemental material

- Supplemental file 1 -

  Fig. S1 to S3 and Tables S1 and S2

  PDF, 522K
