# Supplementary material for: Age, Weight, and CYP2D6 Genotype Are Major Determinants of Primaquine Pharmacokinetics in African Children
Source: Antimicrob Agents Chemother. 2017 Apr 24;61(5):e02590-16. doi: 10.1128/AAC.02590-16 (PMC5404566; doi:10.1128/AAC.02590-16)
Supplement: Supplemental material [file AAC.02590-16_zac005176174so1.pdf]

Supplemental Material

Title: Age, weight, and *CYP2D6* genotype are major determinants of primaquine pharmacokinetics in African children

Authors: Bronner P. Gonçalves, Helmi Pett, Alfred B. Tiono, Daryl Murry, Sodiomon Sirima, Mikko Niemi, Teun Bousema, Chris Drakeley, Rob ter Heine

Figures

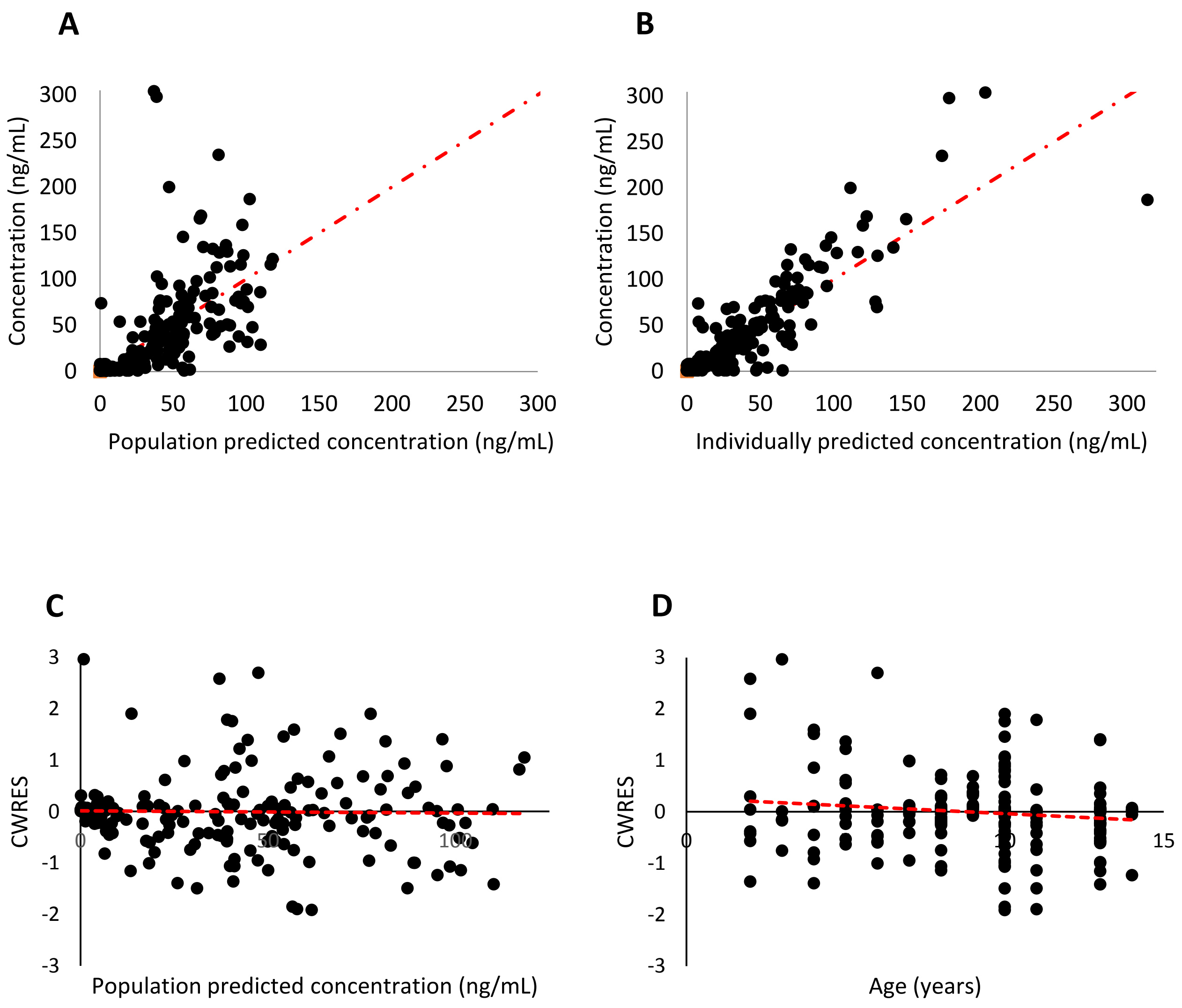

Figure S1. Goodness-of-fit plots of plasma PQ concentrations. In Panel A, population predicted concentrations (x-axis) are compared with observed concentrations (y-axis); in B, individually predicted PQ concentrations are used. Panels C and D present conditional weighted residuals by predicted concentrations and age, respectively.

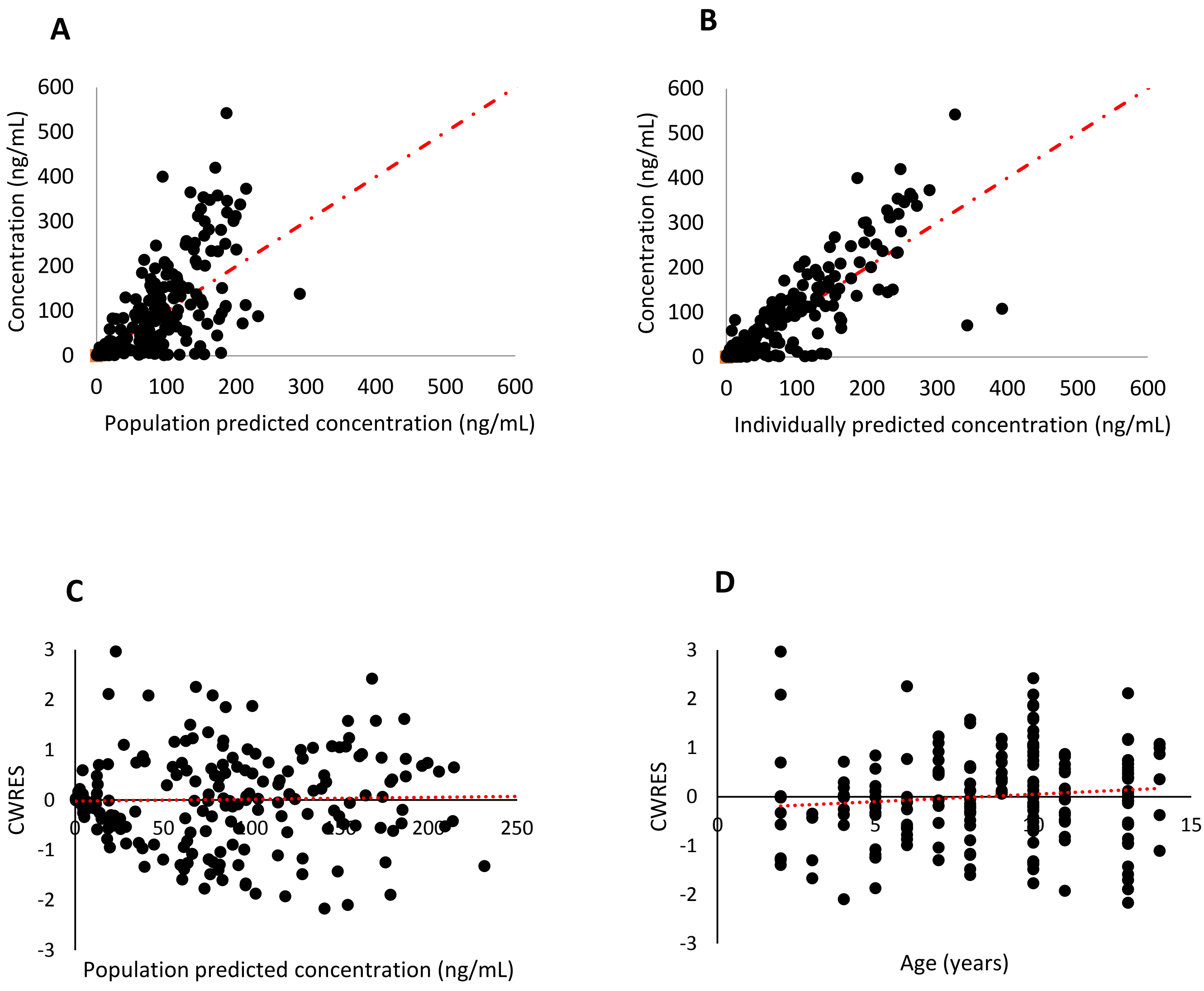

Figure S2. Goodness-of-fit plots of plasma C-PQ concentrations. In Panel A, population predicted concentrations (x-axis) are compared with observed concentrations (y-axis); in B, individually predicted C-PQ concentrations (x-axis) are used. Panels C and D present conditional weighted residuals by predicted concentrations and age, respectively.

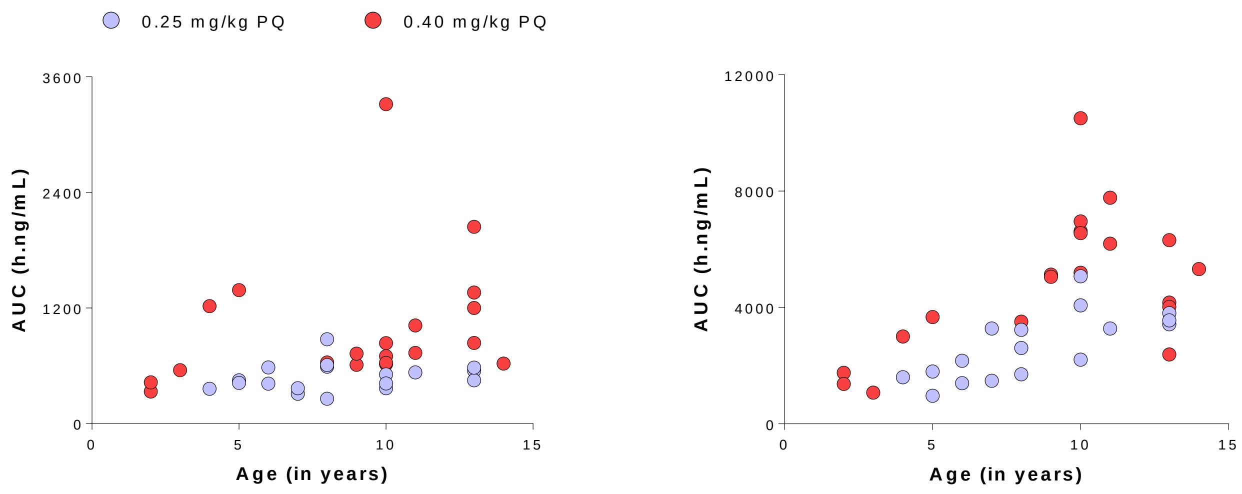

Figure S3. Area under the plasma concentration – time curve for both PQ (left panel) and C-PQ (right panel) by age (x-axes).

Tables

Table S1. TaqMan® OpenArray® PGx Assays (QuantStudio™ 12K Flex)

**Assay ID**	**SNP ID**	**Gene Symbol**	**Allele(s)**	**Nucleotide change**
C__27102425_10	rs16947	CYP2D6	*2, *17, *29, *40 and *41	2850C>T
C__27102414_10	rs1135840	CYP2D6	*2, *4, *10, *17, *29, *40 and *41	4180G>C
C__32407232_50	rs35742686	CYP2D6	*3	2549delA
C__27102431_D0	rs3892097	CYP2D6	*4	1846G>A
C__32407243_20	rs5030655	CYP2D6	*6	1707T>del
C__32388575_30 or C__32388575_A0†	rs5030867	CYP2D6	*7	2935A>C
C_30634117C_20 or C_30634117C_K0†	rs5030865	CYP2D6	*8	1758G>T
C_30634117D_30 or C_30634117D_M0†	rs5030865	CYP2D6	*14	1758G>A
C__32407229_60	NULL	CYP2D6	*9	2613_2615delAGA
C__11484460_40	rs1065852	CYP2D6	*10 and *4	100C>T
C__30634118_A0	rs5030863	CYP2D6	*11	883G>C
C__32407245_40‡	rs72549357	CYP2D6	*15	137-138insT
C___2222771_40 or C___2222771_A0†	rs28371706	CYP2D6	*17 and *40	1023C>T

C__32407220_60	NULL	CYP2D6	*18	4125_4133dupGTGCCCACT
C__32407233_50	rs72549353	CYP2D6	*19	2539_2542delAACT
C__72649949_10	rs72549354	CYP2D6	*20	1973_1974insG
C__34816113_20	rs59421388	CYP2D6	*29	3183G>A
C__32407240_80	NULL	CYP2D6	*40	1863_1864insTTTCGCCCC
C__34816116_20	rs28371725	CYP2D6	*41	2988G>A

†A new assay was developed at Thermo Fisher for these SNPs during this study.

‡Results of this assay were disregarded as it was later determined to be amplifying also from pseudogene *CYP2D7*. (Riffel et al. CYP2D7 Sequence Variation Interferes with TaqMan CYP2D6 (*) 15 and (*) 35 Genotyping. Front Pharmacol. 2016)

Table S2. *CYP2D6* allele frequencies

Haplotype	100	1023	1846	2850	3183	4180	Duplication	Exon 9	Enzyme Activity	Heterozygotes	Homozygotes	Allele Frequency
*1	C	C	G	C	G	G	NO	YES	Normal	16	3	0.31
*2	C	C	G	T	G	C	NO	YES	Normal	9	1	0.15
*2x2†	C	C	G	T	G	C	YES	YES	Increased	3	0	0.04
*4	T	C	A	C	G	C	NO	YES	None	1	0	0.01
*5	-	-	-	-	-	-	NO	NO	None	5	1	0.10
*10	T	C	G	C	G	C	NO	YES	Decreased	1	0	0.01
*17	C	T	G	T	G	C	NO	YES	Decreased	15	1	0.24
*17x2†	C	T	G	T	G	C	YES	YES	?	2	0	0.03
*29	C	C	G	T	A	C	NO	YES	Decreased	7	0	0.10
*36	T	C	G	C	G	C	NO	NO	Negligible	1	0	0.01

100, 1023, 1846, 2850, 3183, 4180 are SNP/indel locations on gene according to www.cypalleles.ki.se.

†Duplications may go undetected with genotypes *2x2/*5 and *5/*17x2, as these will be genotyped as *2/*2 and *17/*17 respectively.
